# Combined Effects of Diosmin, Hesperidin, *Ruscus aculeatus*, *Ananas comosus*, and Bromelain on Endothelial Function and Gut Barrier Integrity In Vitro

**DOI:** 10.3390/ijms262110538

**Published:** 2025-10-29

**Authors:** Rebecca Galla, Simone Mulè, Sara Ferrari, Claudio Molinari, Francesca Uberti

**Affiliations:** 1 Noivita S.r.l.s., Spin Off of University of Piemonte Orientale, Strada Privata Curti n. 7, 28100 Novara, Italy; 2 Laboratory of Physiology, Department for Sustainable Development and Ecological Transition, University of Piemonte Orientale, UPO, 13100 Vercelli, Italy

**Keywords:** venous insufficiency, intestinal absorption, endothelial cell, flavonoids

## Abstract

The endothelium, once considered merely a vascular lining responsible for selective permeability to water and electrolytes, is now recognised as a key regulator of vascular tone through the release of mediators such as oxylipins, nitric oxide, and hyperpolarizing factors. This in vitro study investigated the biological activity of Vesvein, a natural formulation containing Diosmin/Hesperidin, *Ruscus aculeatus*, Bromelain, and *Ananas comosus*, on intestinal and endothelial cells. Vesvein enhanced intestinal cell viability and preserved barrier integrity, as demonstrated by increased tight junction expression at both single and double concentrations. In endothelial cells, the compound improved parameters linked to venous insufficiency, elevating nitric oxide production by approximately 1.39-fold at a single dose and 1.65-fold at a double dose. These findings indicate a potential role for Vesvein in supporting endothelial health and vascular function in vitro. Preliminary evidence from intestinal models further suggests preserved barrier properties, which may positively influence absorption and bioavailability, thereby enhancing its vascular benefits.

## 1. Introduction

Endothelial cells (ECs) are crucial to maintaining health and preventing disease by regulating blood vessel function, inflammation, oxidation and coagulation through specific receptors on their surface. Alterations in these receptors can cause inflammation, increased cell adhesion, abnormal cell growth and blood clotting [1].

The endothelium is involved in several homeostatic functions, mediated by membrane-bound receptors for various molecular entities, including proteins, lipid transport vesicles, metabolites, hormones, and junctional receptors [2]. The endothelium influences the tone of blood vessels by producing and secreting vasodilating substances such as nitric oxide (NO), which are essential for maintaining optimal vascular tone under pathological conditions [3]. Indeed, reduced NO bioavailability promotes smooth muscle cell activation and immune cell recruitment, which in turn affects endothelial cell dysregulation of vascular tone. Chronic venous illness is made worse by this dysfunction, which prolongs the inflammatory cascade and causes pathological alterations in the veins [4,5].

About 5 to 33% of adults suffer from chronic venous disease (CVD) of the lower extremities. Patients may experience benign symptoms or more severe clinical manifestations, such as distal venous hypertension, painful oedema, swelling and venous ulcers. Conventional treatments for CVD, such as anticoagulants and compression devices, aim to relieve symptoms without restoring valve function [6]. Among women, cardiovascular disorders and varicose veins (VVs) are more common than among men. Indeed, it is predicted that 17% of men and 40% of women will experience venous insufficiency as they age, due to increased vascular pressure in the lower extremities [7]. People who have venous hypertension are more likely to acquire cardiovascular diseases. Venous reflux and malfunctioning venous valves are the most common causes of this condition, while previous venous blockage may also contribute to its development. In healthy people, contraction of the calf muscles facilitates blood propulsion from the lower extremities [8]. Conversely, insufficient contraction of these muscle groups can impede venous blood flow, thereby inducing vascular and inflammatory processes that intensify hypertension [9]. The aetiology of this pathological condition may be attributed to venous hypertension and venous stasis, resulting in a microcirculatory imbalance and reduced tissue oxygenation [10]. A growing body of evidence supports the idea that cardiovascular disease is an inflammatory disorder influenced by blood pressure. Increased venous pressure creates an atypical biomechanical environment in the venous system, characterised by hemodynamic and biomechanical irregularities that can lead to endothelial dysfunction, ultimately resulting in the premature release and activation of enzymes responsible for degrading the extracellular matrix [11]. According to recent research, increased matrix metalloproteinases (MMPs) can alter the structure of vein walls. Numerous factors, such as venous pressure and inflammation, impair the function of MMPs by degrading crucial extracellular matrix (ECM) proteins and weakening the vein wall. Furthermore, MMPs have the ability to influence proliferation, migration, development, and death of vascular smooth muscle cells (VSMCs) [12]. Although hypertrophic VVs exhibit significant ECM accumulation and abnormal VSMC shape and alignment, in atrophic VVs, there is significant ECM disruption and infiltration of inflammatory cells into the tissue [13]. VV tissue may also show focal thickening of the intimate tunica and increased thickening of the tunica media with fragmented elastin fibres [14]. Vascular endothelial growth factor (VEGF) plays a crucial role in maintaining the structural integrity of blood vessels during the process of angiogenesis. It has been demonstrated that this process can weaken vessel walls by increasing permeability and activating endothelial nitric oxide synthase (eNOS), resulting in blood vessel dilation [15,16].

Some naturally derived substances, such as flavonoids, have been proposed to stimulate endothelial cells to release NO by mediating the increase in intracellular calcium levels and causing immediate vasodilation [10]. For instance, Diosmin, frequently used in combination with Hesperidin (another flavonoid), is widely employed in managing CVD. This combination enhances venous tonicity, diminishes capillary permeability, augments lymphatic drainage, and exhibits anti-inflammatory properties [17]. Empirical investigations have demonstrated that administering micronised flavonoids, such as micronised purified flavonoid fraction (MPFF), markedly alleviates symptoms associated with CVD, including pain, oedema, and a sensation of heaviness in the lower extremities [18]. The micronisation process enhances bioavailability, thereby rendering therapeutic interventions more efficacious even at reduced dosages [19].

*Ruscus aculeatus* L. (Asparagaceae), which encompasses active compounds such as ruscogenin, neuroruscogenin, and various other saponins, has been utilised for an extensive duration in the form of a hydroalcoholic extract to alleviate sensations of heaviness and oedema in the lower extremities. Among the documented pharmacological activities of this extract, one of its primary actions is vasoconstrictive activity, attributed to the agonistic interaction with α-1 and α-2 adrenergic receptors located in the vascular wall, as well as the facilitation of norepinephrine release from adrenergic nerve terminals [20]. Furthermore, existing literature indicates that *Ruscus aculeatus* extract has a significant effect on venous structures, lymphatic vessels, and capillary networks [21]. Specifically, a variety of mechanisms have been proposed to elucidate the protective effects of *Ruscus aculeatus* extract on microcirculation, which include vasoconstriction of vascular structures leading to mitigated venous hypertension and localised protective effects associated with the safeguarding of endothelial cells and anti-inflammatory characteristics [22]. A noteworthy advantage of using *Ruscus aculeatus* extract is its ability to inhibit the augmented permeability of the vascular wall, a process mediated by histamine [23].

Finally, the molecules under study include *Ananas comosus* extract with Bromelain, also used for treating CVD. Bromelain reduces inflammation and oedema by modulating proinflammatory cytokines and improving microcirculation [24]. Bromelain constitutes a complex amalgamation of proteolytic enzymes, primarily extracted from the stem and fruit of the pineapple [25]. It exhibits notable anti-inflammatory and anti-edematous characteristics. These attributes are investigated by activating pathways that facilitate the degradation of inflammatory proteins and mitigate fibrin accumulation within compromised venous structures, thereby enhancing lymphatic drainage and microcirculation [26].

Diosmin and hesperidin, as flavonoids, have demonstrated potent anti-inflammatory activity [27]; it has been reported that their combination may help suppress the production of NO, prostaglandin E2 (PGE2), and tumour necrosis factor α (TNF-α) in lipopolysaccharide (LPS)-activated macrophages [27,28]. Comparison with conventional anti-inflammatory drugs suggests that the combination of Diosmin with hesperidin may have comparable activity in inhibiting inflammatory processes [29]. In addition, Diosmin has a favourable toxicity profile with low levels of toxicity and protective effects. At the same time, hesperidin can reduce the production of NO, PGE2, TNF-α, and interleukin (IL)-6 in inflammatory models [30]. Furthermore, due to its anti-inflammatory effects and low cytotoxicity, hesperidin is a promising agent for therapeutic use, even in combination with Diosmin to improve venous tone, inflammation, and alleviate symptoms of CVD [31].

In addition to this, the presence of *Ruscus aculeatus* is essential as it contains saponins with vasoconstrictor and lymphonotonic activity [32]. Most of the evidence currently available focuses on the evaluation of a combination of *Ruscus aculeatus* extract, hesperidin methyl chalcone (HMC) and ascorbic acid, which exerted positive effects [21,23]. In another study, *Ruscus aculeatus* was combined with a mixture of plant extracts that is supposed to inhibit the activity of activator protein 1 (AP-1), to create a formulation effective against inflammation related to cardiovascular diseases. The efficacy of this preparation was tested on a model of human endothelial cells subjected to severe inflammatory stimulation. The results obtained demonstrated the ability of this preparation to counteract the release of cytokines and the inflammation-induced activation of NF-κB and AP-1 [33].

Lastly, bromelain and *Ananas comosus* extracts, which possess anti-edematous and anti-inflammatory properties, are able to modulate the production of cytokines and enhance microcirculation [25,34]. In the context of cardiovascular diseases, combinations of the enzymes trypsin and bromelain, along with the bioflavonoid rutoside, have proven effective and have been available in many countries for decades. This combination is available in several enteric-coated formulations such as Disperzyme or Phlogam [35].

In light of the scientific evidence previously discussed, the present study sought to investigate the potential of Diosmin/Hesperidin, *Ruscus aculeatus*, Bromelain and *Ananas comosus* to modulate inflammation, oxidative stress, endothelial dysfunction and venous tone, through a complementary effect of their mechanisms. This approach aims to provide a comprehensive strategy for the management of CVD.

## 2. Results

### 2.1. Safety Evaluation of Vesvein and Individual Agents on Intestinal Epithelium to Exclude Toxicity

**Figure 1:**
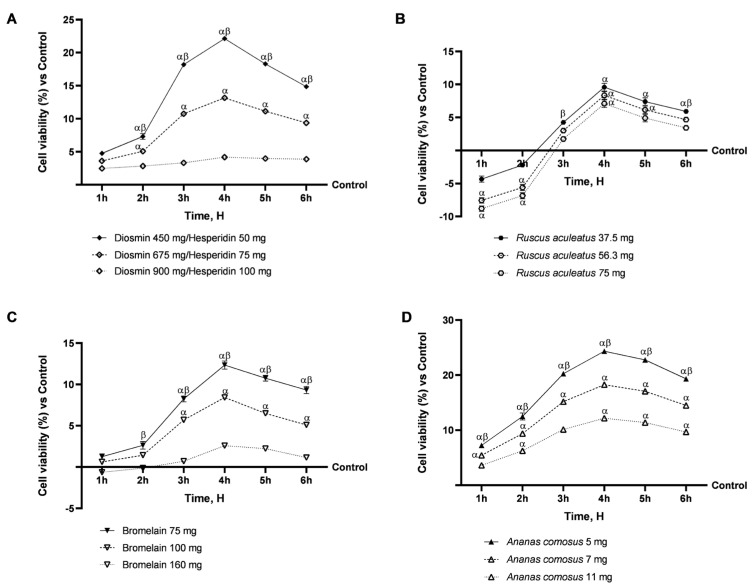
Dose–response study of individual agents on the intestinal epithelium. In (**A**), screening analysis of Diosmin/Hesperidin was performed from 1 h to 6 h using the MTT test; in (**B**), screening analysis of *Ruscus aculeatus* was performed from 1 h to 6 h using the MTT test; in (**C**), screening analysis of Bromelain was performed from 1 h to 6 h using the MTT test; in (**D**), screening analysis of *Ananas comosus* was performed from 1 h to 6 h using the MTT test. Data are expressed as mean ± SD (%) of 5 independent experiments, performed in triplicate, normalised to the control (0%) line. *α p* < 0.05 vs. Control (line 0); *β p* < 0.05 vs. other concentration.

As an initial step, it became crucial to determine whether all the natural extracts being analysed, both singularly and collectively, could maintain intestinal health for a treatment period ranging from 1 to 6 h to rule out any potential cytotoxic effects. The intestinal tests were performed over a time range of 1 to 6 h, as this represents the timeframe for absorption and emptying of the intestine in vivo. The interval of 1–6 h was intentionally chosen based on the physiological time of digestion and intestinal absorption [36]. A range of concentrations of Diosmin/Hesperidin, *Ruscus aculeatus*, Bromelain, and *Ananas comosus* were tested to determine the most effective concentrations to include in the final mix. The concentration and dosage screening is reported in Figure 1, and the data obtained from this screening revealed that the lowest concentrations were the most effective. Indeed, among the various concentrations of Diosmin/Hesperidin, the most effective was identified in 450:50 mg (human dosage) in comparison to the other two dosages of 675:75 mg and 900:100 mg (*p* < 0.05), particularly at 4 h of treatment, achieving approximately 40.5% and 81%, respectively (Figure 1A). Similarly, for *Ruscus aculeatus* and Bromelain, the optimum concentration was determined to be 37.5 mg for the former and 75 mg for the latter, in comparison to the higher concentrations tested (*p* < 0.05). Specifically, *Ruscus aculeatus* 37.5 mg demonstrated a statistically significant effect in comparison to the other two concentrations, exhibiting approximately 29% vs. *Ruscus aculeatus* 56.3 mg (*p* < 0.05) and 59% vs. *Ruscus aculeatus* 75 mg (*p <* 0.05) at the 2 h treatment point, respectively (Figure 1B). Conversely, Bromelain 75 mg exhibited a beneficial effect that exceeded approximately 32% vs. Bromelain 100 mg (*p <* 0.05) and 79% vs. Bromelain 160 mg (*p <* 0.05), following a 4 h treatment duration (Figure 1C). Finally, *Ananas comosus* also demonstrated a greater beneficial effect on cell viability at the concentration of 5 mg compared to the other two concentrations of 7 mg (*p <* 0.05) and 11 mg (*p <* 0.05), with a peak effect at 4 h of approximately 25% and 50%, respectively (Figure 1D). Consequently, the following concentrations were utilised in subsequent experiments: Diosmin 450 mg/Hesperidin 50 mg, *Ruscus aculeatus* 37.5 mg, Bromelain 75 mg and *Ananas comosus* 5 mg.

**Figure 2:**
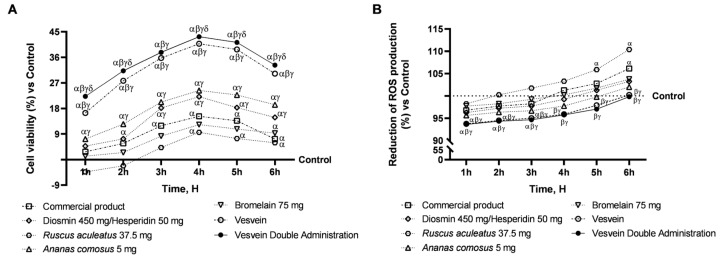
Safety evaluation of Vesvein and individual agents on the intestinal epithelium to exclude toxicity. In (**A**), cell viability analysis was performed from 1 h to 6 h using the MTT test; in (**B**), the ROS production was analysed from 1 h to 6 h using Cytochrome C reduction. Vesvein = Diosmin/Hesperidin + *Ruscus aculeatus* + *Ananas comosus* + Bromelain; Vesvein Double Administration = Vesvein combination administered twice for each point of treatment. Data are expressed as mean ± SD (%) of 5 independent experiments, performed in triplicate, normalised to the control (0%) line. *α p* < 0.0001 vs. Control (line 0); *β p* < 0.0001 vs. single agents; *γ p* < 0.0001 vs. commercial product; *δ p* < 0.0001 vs. Vesvein.

Consequently, based on the initial screening, a cell viability assay and an assessment of reactive oxygen species (ROS) production at the intestinal level were conducted to substantiate the safety profile of the samples under scrutiny and their combination. More specifically, Diosmin/Hesperidin, *Ruscus aculeatus*, Bromelain, and *Ananas comosus* extract were examined at the intestinal level, both separately and in combination (referred to as Vesvein), and their effects were compared to those of a commercially available product. Furthermore, the Vesvein combination was also evaluated at a double dosage to replicate the effects of double administration (Vesvein Double Administration). As shown in Figure 2A, all individual agents increased cell viability, with a peak effect at 4 h of treatment compared with the control (untreated cells, 0% line, *p* < 0.05) and aligning their effect with that exerted by the commercial product. In contrast, the combination was able to increase cell viability compared with the control and single agents (*p* < 0.0001) but also outperformed the commercial product in effect at 4 h of treatment by about 63% (*p* < 0.0001). The findings suggest that the combination of substances may enhance their overall effect, as co-administration appears to amplify the effect relative to when administered individually. In addition, the Vesvein combination administered a double dose to emulate double administration showed greater beneficial effects than the control and single agents, with a maximum effect of Vesvein double-dose at 4 h of about 6% compared to Vesvein (*p* < 0.05) and about 65% compared to the commercial product (*p* < 0.0001). To summarise, the viability data showed that Vesvein Double Administration was safe for intestinal cells. Similarly, the same trend was observed in ROS production (Figure 2B). Specifically, all individual agents can maintain ROS levels below the control value (1 h to 4 h of treatment) or below the threshold values (5 h to 6 h). The effects exerted by the individual agents were equal to those exerted by the commercial product. In contrast, the most significant beneficial effect was observed after single or double administration of Vesvein compared with the individual agents and the commercial product (*p* < 0.05), with no significant difference between the two different administrations.

### 2.2. Integrity and Permeability Analysis on a 3D Model of the Intestinal Barrier In Vitro

**Figure 3:**
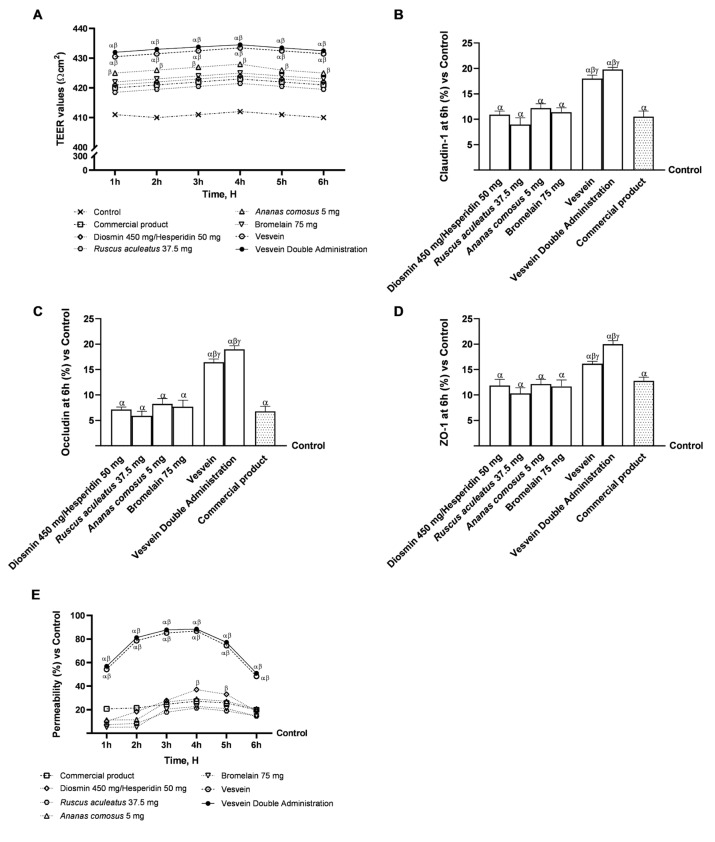
Integrity and permeability analysis on a 3D model of the intestinal barrier in vitro. In (**A**), TEER Value measurement using EVOM3 during the time (from 1 h to 6 h); from (**B**–**D**), the analysis of TJ measured by Enzyme-Linked Immunosorbent Assay (ELISA) test (Occludin, Claudin-1, and ZO-1, respectively) at 6 h; in (**E**), permeability (from 1 h to 6 h) measured with fluorescein on the intestinal barrier. In (**A**,**E**), data are expressed as means ± SD of five independent experiments performed in triplicate. All substances were *p* < 0.05 vs. Control (line 0); *α p* < 0.0001 vs. single agents; β *p* < 0.0001 vs. commercial product. From (**B**–**D**), means ± SD were expressed by comparing data to the control (0% line) of five independent experiments performed in triplicate. *α p* < 0.05 vs. Control (line 0); *β p* < 0.05 vs. single agents; *γ p* < 0.05 vs. commercial product.

To evaluate the permeability and transport of Vesvein, Vesvein Double Administration, and individual components, further experiments were performed on an in vitro 3D model of the intestinal barrier, mimicking as closely as possible what happens in vivo. In this context, all test substances were tested from 1 h to 6 h to measure transepithelial electrical resistance (TEER) values and absorption rate. In addition, TJ protein levels were assessed to confirm the integrity of the intestinal monolayer following treatment with all the agents under investigation. Specifically, as shown in Figure 3A, the TEER values obtained during the treatment period demonstrated that both single agents and their combinations were able to maintain TEER values above those of the control. In addition, Vesvein and Vesvein Dual Administration were observed to be more effective than the single agents and the reference product (*p <* 0.0001), suggesting a more favourable modulation of paracellular efflux and ion transport through the intestinal epithelium. These results were confirmed by evaluating the levels of TJ proteins, including Claudin-1, Occludin, and ZO-1. Indeed, all individual agents evaluated were able to increase the levels of all TJs under analysis compared to the control, with effects comparable to those of the commercial product (Figure 3B–D). Conversely, when the individual agents were combined, the resultant effect was magnified: specifically, Vesvein and Vesvein Double Administration enhanced Claudin-1 levels by approximately 39% and 45% compared to Diosmin/Hesperidin (*p* < 0.0001), 50% and 55% compared to *Ruscus aculeatus* (*p* < 0.0001), 32% and 38% compared to *Ananas comosus* (*p* < 0.0001), 37% and 42% compared to Bromelain (*p* < 0.0001), and about 41% and 47% compared to the commercial product (*p* < 0.0001), respectively. Additionally, Occludin levels were also significantly increased following treatment with Vesvein and Vesvein Double Administration by approximately 56.5% and 62% compared to Diosmin/Hesperidin (*p* < 0.0001), 64% and 69% compared to *Ruscus aculeatus* (*p* < 0.0001), 53% and 59% compared to *Ananas comosus* (*p* < 0.0001), 50% and 57% compared to Bromelain (*p* < 0.0001), and about 59% and 64% compared to the commercial product (*p* < 0.0001). Lastly, Vesvein and Vesvein Double Administration are further capable of elevating ZO-1 levels by approximately 26.5% and 41% vs. Diosmin/Hesperidin (*p* < 0.0001), 36% and 48% vs. *Ruscus aculeatus* (*p* < 0.0001), 25% and 39% vs. *Ananas comosus* (*p* < 0.0001), 27.5% and 41.5% vs. Bromelain (*p* < 0.0001), and by approximately 21% and 36% vs. commercial product (*p* < 0.0001). Furthermore, an analysis of the permeability (Figure 3E) was conducted for all agents investigated. Again, Vesvein and Vesvein double administration exhibited a higher rate of absorption compared to the single agents and the commercial product (*p <* 0.0001).

### 2.3. Effect of Individual Components and Combinations on the 3D Vein Model

**Figure 4:**
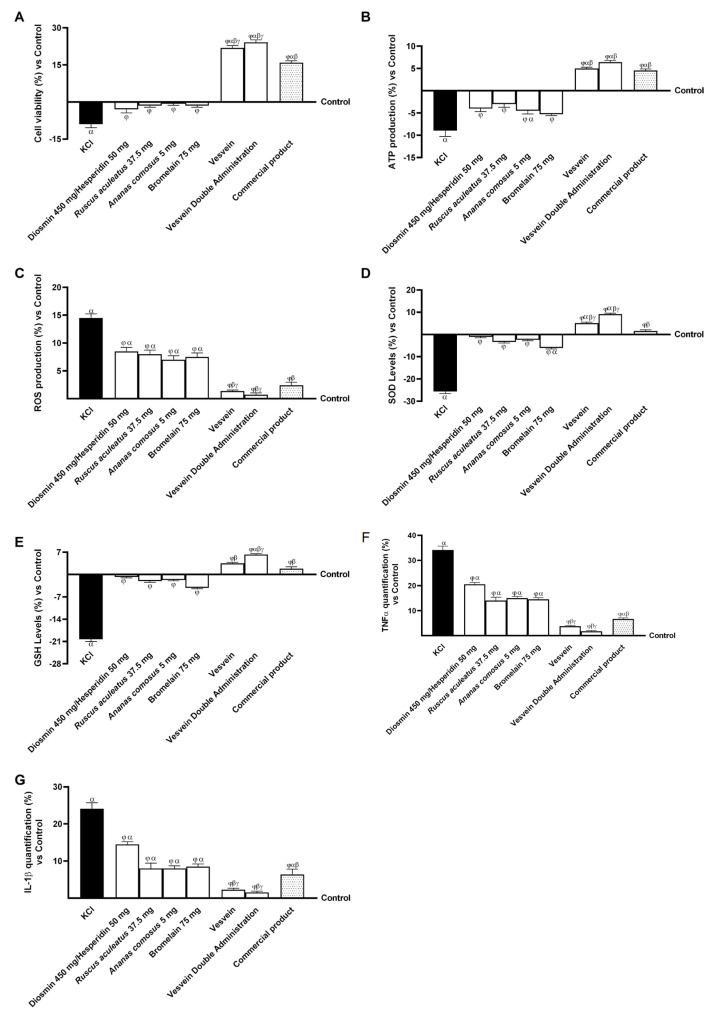
Effect of individual components and combinations on the 3D vein model. In (**A**), analysis of cell viability by MTT test; in (**B**), analysis of ATP production by specific Kit; in (**C**), analysis of ROS production by Cytochrome C reduction; in (**D**), SOD levels determination by ELISA Kit; in (**E**), GSH levels analysed by ELISA Kit; in (**F**), TNF-α production analysis by ELISA Kit; and in (**G**), IL-1β production analysis by ELISA Kit. Vesvein = Diosmin/Hesperidin + *Ruscus aculeatus* + *Ananas comosus* + Bromelain; Vesvein Double Administration = Vesvein combination administered twice for each point of treatment. Data are expressed as mean ± SD (%) of 5 independent experiments, performed in triplicate, normalised to the control (0%) line. *α p* < 0.05 vs. Control (line 0); *β p*< 0.05 vs. single agents; *γ p* < 0.05 vs. commercial product; *φ p* < 0.05 vs. KCl 96 mM.

**Figure A1:**
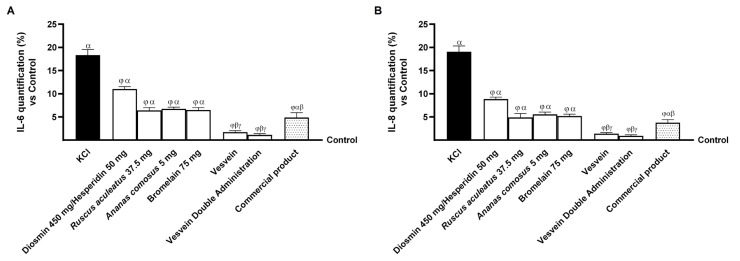
Quantification of (**A**) IL-6 and (**B**) IL-8 in HUVEC cell lysate using specific ELISA Kit. *α p* < 0.05 vs. Control (line 0); *β p* < 0.05 vs. single agents; *γ p* < 0.05 vs. commercial product; *φ p* < 0.05 vs. KCl 96 mM.

Based on the data collected at the gut level, further experiments were conducted using a three-dimensional vascular model. To replicate the characteristics associated with CVD in vitro, HUVECs, used in the 3D vascular model, were treated for 15 min after exposure to a specific inducer (96 mM KCl), which activates the mechanisms of ECM degradation and increases venous pressure [10]. As shown in Figure 4, all agents under investigation were able to restore cell viability and reduce oxidative stress by counteracting the negative effects of KCl (*p* < 0.05). In particular, the effects of individual agents and combinations on the KCl-induced CVD condition are shown in Figure 4A. After damage with KCl, the viability of venous cells was drastically reduced compared to the control (untreated cells, *p* < 0.05). However, cells treated with the single agents were able to resist the induced damage with a significant increase in cell viability (*p* < 0.05). The combination showed a significant protective effect compared to single agents and commercial product, with increases in viability of approximately 27% (*p <* 0.05) and 34% (*p <* 0.05), respectively. Similarly, the beneficial protective effect of the combinations and their agents was also observed on ATP production (Figure 4B). Specifically, KCl significantly reduced ATP production compared to the control (*p <* 0.05). However, Vesvein and Vesvein Double Administration treatment restored ATP production compared with KCl-induced damage by approximately 1.56-fold and 1.72-fold (*p <* 0.05). In addition, ROS production was evaluated to assess the antioxidant effect of the individual agents and their combinations against KCl-induced damage. In particular, as a result of KCl-induced damage, the production of ROS was significantly increased (approximately 14.5%) compared to the control (*p <* 0.05); surprisingly, cells treated with the single agents were able to resist induced damage by significantly reducing ROS production (Figure 4C). However, compared with single agents, the most significant benefit was observed after treatment with Vesvein and Vesvein Double Administration. In order to corroborate the observations on ROS production, the levels of SOD and GSH were also investigated after damage induction and after treatment with all the agents under examination. As demonstrated in Figure 4D,E, KCl-induced damage was found to reduce the levels of the two enzymes involved in reducing oxidative stress (SOD and GSH), resulting in an increase in free radicals in comparison to the control (*p* < 0.05). In contrast, treatment with individual agents was found to restore the levels of these two enzymes to almost control levels, thereby increasing them compared to the damage (*p* < 0.05). Specifically, following treatment with Vesvein and Vesvein Double Administration, significant increases in SOD and GSH levels were observed, exceeding the control values when compared to the individual agents and damage (*p* < 0.05). Moreover, both treatments were found to exceed the effect of the commercial product by approximately 70% and 83% on SOD levels (*p* < 0.05) and by approximately 47% and 70% on GSH levels (*p* < 0.05). Finally, to confirm the beneficial effect of all the agents under investigation, the inflammatory response of cells to KCl-induced damage was analysed by measuring the production of TNF-α (Figure 4F) and interleukin (IL)-1β (Figure 4G). Specifically, the individual agents and the commercial product were able to reduce the release of inflammatory cytokines to levels below those produced by damage with KCl (*p <* 0.05). The most significant beneficial effects on reducing TNF-α and IL-1β production were observed after treatment with Vesvein and Vesvein Double Administration, as they were able to reduce TNF-α production by approximately 89% and 95%, respectively, compared with KCl (*p <* 0.05). In comparison, IL-1β production was decreased by approximately 90.5% and 94% compared with KCl (*p <* 0.05). Furthermore, in order to confirm these results, the production of IL-6 and IL-8 was also investigated (see Figure A1 in the Appendix A). The data corroborate the observations made for the other two parameters, as Vesvein and Vesvein Double Administration were found to be more efficacious in reducing the inflammatory response than the individual agents (*p* < 0.05). Furthermore, Vesvein and Vesvein Double Administration demonstrated a more pronounced beneficial effect in comparison to the competitor on both parameters, with a reduction of approximately 64% and 63% (Vesvein vs. commercial product, IL-6, *p* < 0.05) and approximately 76.5% and 75% (Vesvein Double Administration vs. commercial product, IL-8, *p* < 0.05).

These data support the initial hypothesis that the Vesvein Double Administration combination can maintain the well-being of venous cells even once placed in a condition of venous insufficiency by KCl treatment.

### 2.4. Effect of Individual Components and Combinations on Maintaining Venous Integrity and Tone

**Figure 5:**
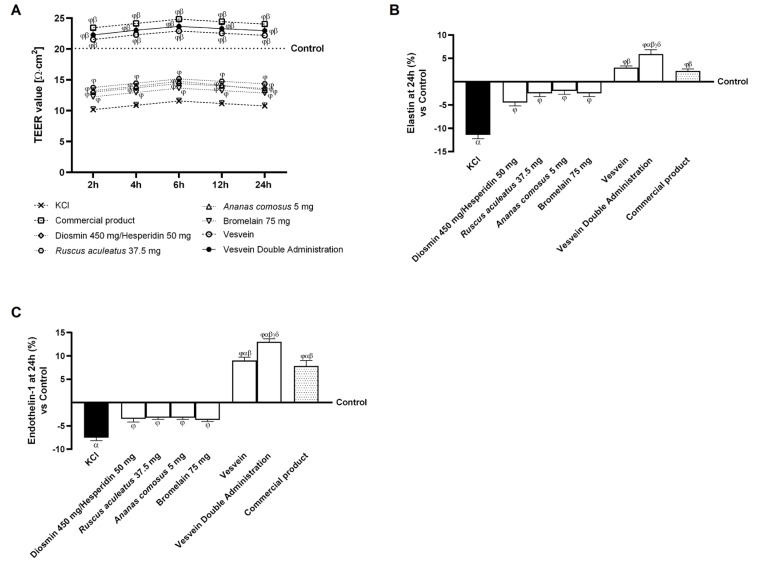
Effect of individual components and combinations on the 3D vein model. In (**A**), TEER Value measurement using EVOM3 during the time (from 2h to 24h); in (**B**), quantification analysis of Elastin levels by specific Kit; and in (**C**), quantification analysis of Endothelin-1 levels by specific Kit. Vesvein = Diosmin/Hesperidin + *Ruscus aculeatus* + *Ananas comosus* + Bromelain; Vesvein Double Administration = Vesvein combination administered twice for each point of treatment. Data are expressed as mean ± SD (%) of 5 independent experiments, performed in triplicate, normalised to the control (0%) line. In (**A**), *β p* < 0.05 vs. single agents; *φ p* < 0.05 vs. KCl. From (**B**,**C**), *α p* < 0.05 vs. Control (line 0); *β p* < 0.05 vs. single agents; *γ p* < 0.05 vs. commercial product; *δ p* < 0.05 vs. Vesvein; *φ p* < 0.05 vs. KCl 96 mM.

Once the primary beneficial effects of the individual agents and their combinations on cell viability, ROS production, and pro-inflammatory cytokines were determined, further analyses were carried out to assess vascular integrity, the regulation of vascular contraction and relaxation mechanisms, neovascularisation, vascular permeability, and vasodilation. The TEER value was quantified in cells treated with all agents and placed under damage conditions with KCl (Figure 5A). The data revealed that individual agents were able to increase TEER values relative to KCl-induced damage (*p <* 0.05), without restoring them to control levels. In contrast, the combinations of Vesvein and Vesvein Double Administration, as well as the commercial product, demonstrated the ability to increase TEER values even above those of the control (*p <* 0.05). Additionally, these two combinations have demonstrated effectiveness equivalent to that of the commercial product. In addition, to determine whether the agents tested can exert a regulatory effect on vessel contraction and relaxation movements, elastin levels were evaluated after treatment with all the molecules tested and after induction of damage with KCl (Figure 5B). The data showed that KCl reduced elastin levels by approximately 11.4% compared to the control (*p <* 0.05). In contrast, individual agents and their combinations were able to reverse this behaviour by restoring elastin levels to those of KCl (*p <* 0.05). In addition, of the two combinations studied, only the double administration of Vesvein was able to exert a more significant beneficial effect than the commercial product by approximately 61% (*p <* 0.05) and about 49% compared to Vesvein (*p <* 0.05). Finally, Endothelin (ET)-1 was examined as a potential biomarker for neovascularisation by inducing an angiogenic phenotype in the cellular model. As illustrated in Figure 5C, KCl-induced cytotoxicity resulted in a reduction of Endothelin-1 concentrations by approximately 7.5% when compared to the control group (*p* < 0.05); conversely, the administration of Diosmin/Hesperidin, *Ruscus aculeatus*, *Ananas comosus*, Bromelain, Vesvein, Vesvein Double Administration, and the commercial product demonstrated the capacity to elevate Endothelin-1 levels relative to KCl-induced damage by approximately 53%, 56.6%, 57%, 50%, 2.2-fold, 2.7-fold, and 2.04-fold, respectively (*p* < 0.05). Regarding combined effects, only Vesvein Double Administration displayed a significantly more advantageous outcome than the commercial product, enhancing endothelin-1 levels by approximately 40% relative to the commercial product (*p* < 0.05) and by approximately 31% compared to Vesvein. Collectively, these findings indicate a modulatory influence exerted by all agents under scrutiny, particularly highlighting the efficacy of the Vesvein Double Administration combination in regulating the mechanisms governing vascular contraction, relaxation, and neovascularisation.

**Figure 6:**
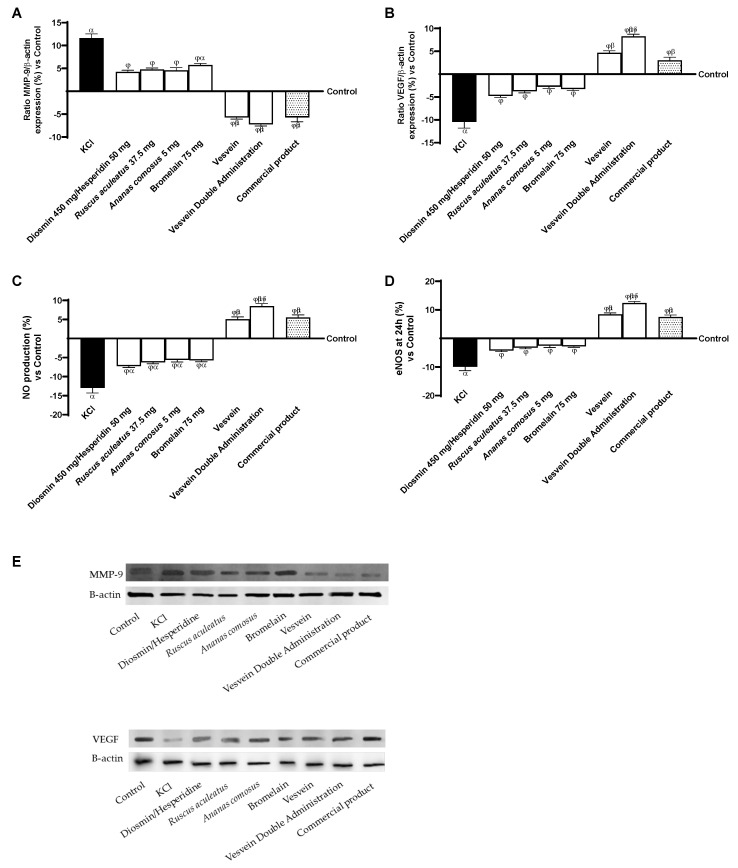
Effect of individual components and combinations on the 3D vein model. In (**A**), MMP-9 expression by Western blot analysis; in (**B**), VEGF expression by Western blot analysis; in (**C**), quantification analysis of NO production by specific Kit; in (**D**), analysis of eNOS levels by specific Kit; and in (**E**), an example of lane from Western blot analysis. Vesvein = Diosmin/Hesperidin + *Ruscus aculeatus* + *Ananas comosus* + Bromelain; Vesvein Double Administration = Vesvein combination administered twice for each point of treatment. Data are expressed as mean ± SD (%) of 5 independent experiments, performed in triplicate, normalised to the control (0%) line. *α p* < 0.05 vs. Control (line 0); *β p* < 0.05 vs. single agents; *γ p* < 0.05 vs. commercial product; *δ p* < 0.05 vs. Vesvein; *φ p* < 0.05 vs. KCl 96 mM.

Finally, since there is a relationship between the expression of MMPs, VEGF, eNOS and the production of NO in the condition of CVD, all these parameters have been analysed following treatment with all agents under investigation. As shown in Figure 6A, KCl-induced damage was able to stimulate the release of MMP-9 by about 11.65% compared to control (*p <* 0.05). In comparison, the individual agents were able to prevent ECM degradation and therefore venous degeneration by reducing levels of MMP-9 compared to the damage induced by KCI. In terms of combinations, Vesvein and Vesvein Double Administration were able to reduce MMP-9 levels better than single agents (2.35 times and 2.76 times compared to Diosmin/Hesperidin, *p <* 0.05; 2.21 times and 2.55 times compared to *Ruscus aculeatus*, *p* < 0.05; 2.25 times and 2.58 times compared to Ananas comosus, *p* < 0.05; twice and 2.26 times compared to Bromelain, *p* < 0.05), showing an effect equivalent to that of the commercial product. As mentioned earlier, VEGF also appears to be an important marker for vascular reactivity, so this was also evaluated following treatment with the agents under study. In Figure 6B, VEGF levels are drastically reduced compared with control (−10.45%, *p* < 0.05) after KCl treatment. VEGF levels were restored after treatment with the individual agents. However, the most significant recovery was observed after treatment with the Vesvein Double Administration, which showed a more substantial effect, approximately 43% and 64% compared with Vesvein and the commercial product (*p <* 0.05). Next, eNOS levels (Figure 6C) and NO production (Figure 6D) were assessed following treatment and induction of damage. The data obtained showed that Vesvein Double Administration was able to result in increased NO production in a better manner than the individual agents (1.86-fold vs. Diosmin/Hesperidin, *p* < 0.05; 1.74-fold vs. *Ruscus aculeatus*, *p* < 0.05; 1.66-fold vs. *Ananas comosus*, *p* < 0.05; 1.68-fold vs. Bromelain, *p* < 0.05), the commercial product (54%, *p* < 0.05) and Vesvein (68%, *p* < 0.05). This increase was found to be a consequence of increased eNOS levels at the cellular level after treatment with the agents under investigation. Specifically, as expected, Vesvein Double Administration increased eNOS levels better than the individual agents (34-fold vs. Diosmin/Hesperidin, *p* < 0.05; 1.26-fold vs. *Ruscus aculeatus*, *p* < 0.05; 1.21-fold vs. *Ananas comosus*, *p* < 0.05; 1.22-fold vs. Bromelain, *p* < 0.05), the commercial product (65%, *p* < 0.05) and Vesvein (48%, *p* < 0.05).

## 3. Discussion

CVD refers to a range of venous diseases which can be caused by dysfunctional valves, an elevated body mass index, pregnancy, gender, age, with recurrence of symptoms within five years after surgery [37]. These conditions contribute to increased intraluminal pressure and venous hypertension, increasing venous wall stress [38]. Leukocyte infiltration and inflammation in the venous wall are the result of damage associated with venous hypertension, which is crucial in the pathogenesis and progression of cardiovascular disease. IL-1β and TNF-α are central mediators in the inflammation cascade [39,40]. CVD may be caused by increased MMP expression, leading to venous wall dilation and inflammatory processes [12]. VEGF, a critical factor in angiogenesis, maintains vascular integrity by mediating NO, vessel permeability, and dilatory responses [10]. It stimulates eNOS expression in endothelial cells, increasing NO formation and influencing the activity of endothelial and inflammatory cells [16].

Nutritional or standard pharmaceutical supplements are available to support the management of CVD as it evolves. They can alleviate the signs and symptoms of CVD as an adjunctive treatment. Furthermore, a registry indicates that patients using adjunctive medical management for CVD benefit from several products [41]. Among them, Diosmin is a flavonoid that has been shown to have inhibitory effects on inflammation and venotonic properties [42]. Furthermore, extracts of *Ruscus aculeatus*, *Ananas comosus*, and Bromelain have been utilised as a natural medicine for chronic venous disease due to their antiedematous, anti-inflammatory, and venotonic effects [23,24]. The present study investigated the effects of a novel formulation, Vesvein, comprising Diosmin/Hesperidin, *Ruscus aculeatus*, *Ananas comosus*, and Bromelain, administered in single and double doses. The preliminary phase of the experiments, conducted at the intestinal level, aimed to determine the optimal concentration for each substance to be used in subsequent experiments. The findings of the present study indicate that the lowest concentration of all the agents under study was the most effective in terms of cellular viability. This approach enabled the selection of concentrations for the production of the final combination. Subsequently, experiments were conducted at the intestinal level to verify whether the molecules under study, administered individually or in combination, could permeate this model without irritating. The results obtained at the intestinal level, as demonstrated by the 3D model, showed that these components can be administered orally. The results of in vitro permeability experiments demonstrated that both combinations, Vesvein and Vesvein Double Administration, were able to permeate the intestinal monolayer. This outcome substantiates the hypothesis that combining individual agents enhances absorption during the physiological time of intestinal digestion and improves their permeability. From a clinical perspective, the ability to achieve high therapeutic concentrations in a short time could translate into improved management of venous disorders or other conditions where rapid intervention is necessary. Furthermore, Vesvein and Vesvein Double Administration demonstrated the capacity to be incorporated into the intestinal epithelium without causing damage by enhancing the expression of TJ proteins. The formation of TJ in epithelial cells is important for the intestinal barrier. This process is mediated by claudin, occludin, and ZO-1 proteins, essential for the proper functioning of the epithelial barrier [43]. These three proteins are particularly important as ZO-1 facilitates the linkage of claudin and occludin to the cytoskeleton, thereby serving as reliable indicators of optimal gut barrier function [44]. Moreover, Vesvein and Vesvein Double Administration were demonstrated to preserve epithelial integrity and facilitate ion exchange across the intestinal barrier, indicating that these formulations can traverse the cell monolayer without exerting a deleterious effect on the epithelium better than the commercial product. It is crucial to acknowledge that, in addition to the biological effects that have been observed, the potential metabolic and intestinal interactions of flavonoid components must also be given due consideration. Diosmin and its active metabolite, diosmetin, have been reported to inhibit various metabolic enzymes, particularly cytochrome P450 isoforms, and drug transporters, although the relevance in vivo appears to be limited. These mechanisms have the potential to affect the metabolism of drugs with narrow therapeutic windows and the absorption of other nutrients [45,46]. In addition, preclinical studies have demonstrated that hesperidin modulates intestinal glucose absorption by inhibiting SGLT1 and GLUT2 transporters, suggesting a possible influence on carbohydrate absorption [47,48,49].

The second phase of this study aimed to assess the capacity of all-natural molecules and their combinations to stimulate the biological activity of venous endothelial cells subjected to conditions of KCl-induced venous insufficiency [10]. As expected, Vesvein and Vesvein Double Administration formulations could stimulate cell viability, induce ATP production, and reduce ROS and pro-inflammatory cytokine production in venous endothelial cells. The endothelium and glycocalyx play a crucial role in sensing stress changes and in the activation of leukocytes, which can cause venous damage and tissue inflammation, leading to a consequent imbalance in pro-inflammatory cytokine production [50]. It should also be noted that the endothelial layer is responsible for maintaining vascular tone and the permeability of the vessel wall, so its impairment could promote adverse events leading to increased vascular tone [14,51]. The endothelium maintains the local balance between pro- and anti-inflammatory factors, and its dysfunction leads to local vascular changes and disturbances of homeostasis [52]. These results support the hypothesis that this combination can be used for treating cardiovascular diseases by targeting inflammatory processes. Indeed, the employment of *Ruscus aculeatus* in conjunction with the flavonoids Diosmin and Hesperidin serves to augment the anti-inflammatory effect of the amalgamation. Several studies in the field of literature have demonstrated the anti-inflammatory properties of Diosmin and Hesperidin about CVD [29,31,53,54,55]. Also, Bromelain and *Ananas comosus* extracts exhibit potent anti-edematous and anti-inflammatory properties, primarily by modulating cytokine production and enhancing microcirculation [34]. Indeed, in recent studies, a wide range of therapeutic benefits has been suggested for bromelain, including anti-inflammatory, anti-edematous, analgesic, wound-healing, and anticoagulant effects [25,35].

Our study showed that the agents under investigation could modulate integrity, elastin and endothelin-1 levels when combined. Specifically, the Vesvein Double Administration restored TEER levels to a higher level than the control, restoring levels altered by KCl damage. Similarly, elastin levels, a marker of vascular contraction and relaxation movements, were also improved compared to KCl damage after treatment with Vesvein Double Administration. Finally, as vascular permeability is altered during the process leading to vascular failure, it was important to assess endothelin-1 levels. This marker was also significantly improved after treatment with Vesvein Double Administration compared to KCl-induced damage. Overall, these data confirm what is already known in the literature about the action of flavonoids in maintaining vascular tone [10], particularly when the effects of the combination under investigation are compared with those of the commercial product. Indeed, Vesvein Double Administration showed better effects on all parameters analysed, confirming its possible application in the treatment of CVD. In addition, the impairment of endothelium-derived NO synthesis associated with endothelial dysfunction causes or increases several adverse effects, including vasoconstriction and thrombosis, as well as the expression of pro-inflammatory cytokines and chemokines [51]. Therefore, these parameters were also analysed in the present study to confirm the previous data. Although KCl-induced damage could impair NO synthesis by reducing eNOS levels, Vesvein Double Administration restored eNOS levels and consequently NO production, reversing the damaging trend of KCl. Vesvein Double Administration showed a greater effect in this context, preventing the decrease in eNOS and enhancing its expression more effectively than the commercial product. Indeed, Vesvein contains flavonoids, a large group of polyphenols that have the capacity to act on NO signalling and metabolism. This results in improved eNOS expression and activity, as well as reduced eNOS uncoupling. In addition, it has been established that polyphenols exert a significant effect on NO-dependent vasodilation [56,57]. Indeed, the literature reported that Diosmin and Hesperidin were able to modulate NO production [30].

Interestingly, a concomitant increase in ET-1 was also observed. Although apparently counterintuitive, this may represent a compensatory feedback mechanism within the endothelium. Elevated ET-1 levels may reflect vascular homeostasis restoration attempts following NO-mediated relaxation, particularly in the context of chronic venous insufficiency [58]. Moreover, oxidative stress and eNOS uncoupling could limit NO bioavailability despite its apparent production, thereby promoting compensatory ET-1 release [59]. ET-1 may also exert endothelial-protective effects through ETB receptor activation, which enhances eNOS-derived NO synthesis, while pathological ETA/ETB imbalance may shift the system toward vasoconstriction and inflammation [60,61]. Altogether, these findings suggest that Vesvein modulates the NO/ET-1 axis to re-establish vascular homeostasis under inflammatory stress [62].

In summary, clinical evidence suggests that the administration of Vesvein Double Administration reduces the expression of MMP-9 and increases the level of VEGF following KCl-induced damage, demonstrating a protective effect against vascular degeneration and promoting a vascular response. Specifically, these effects are exerted by the flavonoids contained in the combination, in particular by Diosmin, which can modulate the levels of MMP-9 and VEGF in cardiovascular pathology [63]. The concomitant reduction in MMP-9 and increase in VEGF observed after Vesvein treatment may reflect the modulation of shared upstream pathways, such as NF-κB, MAPK, or PI3K/Akt, rather than a direct causal link [64]. Natural flavonoids and saponins have been reported to suppress NF-κB activation, thereby reducing MMP-9 expression, while promoting endothelial repair through upregulation of VEGF and eNOS [65,66,67]. This coordinated molecular response could underlie the dual vasoprotective and angiogenic effects observed in our in vitro model.

In conclusion, on all parameters analysed at the venous compartment, only the combination administered as a double dose (Vesvein Double Administration) showed greater beneficial effects than the commercial product, while the combination administered as a single dose (Vesvein) did not outperform the impact of the commercial product on all parameters analysed. However, the new Vesvein combination demonstrated beneficial effects for both administrations in a dose-dependent manner. The Vesvein double-dose formulation, especially, was assessed in the study to model potential patient scenarios in which the formulation could be administered at a higher frequency or dose in the acute phases of venous insufficiency. This methodological approach permitted the evaluation of both dose dependence and potential cumulative effects. It is important to note that both MTT viability assays and ROS production data did not demonstrate any signs of toxicity, even at double the dose, in all of the experimental conditions examined. The safety of this double administration is further supported by extant literature, as evidenced by clinical trials investigating the effects of *Ruscus aculeatus*, Diosmin, Hesperidin and Bromelain in the treatment of CVD [63,68,69,70].

A hypothesis was formulated proposing that the combination of the selected compounds would result in the enhancement of the mechanisms of action of the individual agents. The in vitro data presented herein lend support to this theory, demonstrating that, while not synergistic, agents operate through distinct but convergent pathways to address inflammation, oxidative stress and vascular dysfunction. This multicomponent approach signifies a more comprehensive therapeutic strategy, thus indicating a potential for future clinical applications. However, further studies are needed to evaluate the potential pharmacokinetic interactions and metabolic effects of the flavonoids contained in Vesvein, particularly their influence on glucose absorption and its transporters SGLT1 and GLUT2.

## 4. Materials and Methods

### 4.1. Materials and Chemical Reagents

All substances tested in the study was donated by Alfasigma S.p.A. (Trezzano Rosa, Milan, Italy). Bromelain 2500 GDU/g is derived from the stipules of *Ananas comosus* (L.) and, in the study, 75 mg of Bromelain 2500 GDU/g corresponds to 187.5 GDU (water extraction, particle size of 40 mesh). The *Ruscus aculeatus* L. used corresponds to the dry extract of the root of *Ruscus aculeatus,* titrated at 10% with saponins calculated as ruscogenin (water–ethanol extraction, 8:1 drug extract ratio, particle size of 500 microns). Dry extract of *Ananas comosus* L. stipules titrated at 250 GDU/g Bromelain was used in the study at a concentration of 5 mg of *Ananas comosus* extract corresponding to 1.25 GDU (*Ananas comosus*, 1:15–20 extract/drug ratio, particle size of 50 mesh corresponding to 300 microns). The Vesvein combination consisted of 450 mg Diosmin, 50 mg Hesperidin, 37.5 mg extract of *Ruscus aculeatus* (10% ruscogenin), 5 mg of *Ananas comosus* extract, and 75 mg of Bromelain (2500 GDU/g). Table 1 lists all the agents tested at the various doses. The commercial product used for comparison consists mainly of 500 mg of bioflavonoids (450 mg Diosmin and 50 mg Hesperidin). All chemicals, cell line and reagents used in this study were purchased from Merck Life Science (Rome, Italy), American Type Culture Collection (Manassas, VA, USA), MyBiosource (San Diego, CA, USA), Cusabio Technology LCC (Houston, TX, USA), R&D Systems (Minneapolis, MN, USA), ThermoFisher (Waltham, MA, USA) and Promega (Madison, WI, USA).

### 4.2. Agent Preparation

Substances were diluted 1:2000 to replicate the human dose in vitro [71]. Specifically, the concentrations tested for Diosmin/Hesperidin in vitro were in a range between 739 µM and 370 µM [72,73], corresponding to 900:100 mg to 450:50 mg in humans; in vitro *Ruscus aculeatus* concentrations were in a range between 37.5 µg/mL and 18.75 µg/mL [33], corresponding to 75 mg to 37.5 mg in humans; the concentrations tested for Bromelain in vitro were within a range between 80 µg/mL and 37.5 µg/mL [66], corresponding to 160 mg and 75 mg in humans. Finally, the *Ananas comosus* extract in vitro was tested in a concentration range of 5.5 μg/mL to 2.5 μg/mL [74], corresponding to human dosages between 11 mg and 5 mg. Following the screening stage, the final product was formulated by initially combining 500 mg of micronised bioflavonoids (comprising 450 mg of Diosmin and 50 mg of hesperidin), which were designed to facilitate rapid absorption. Next, bioflavonoids were mixed with 75 mg (187.5 GDU enzyme activity) of Bromelain 2500 GDU/g (from *Ananas comosus* (L.) Merr. jambs), 37.5 mg (of which ruscogenin 3.75 mg) *Ruscus aculeatus L*. (root extract tit. 10% saponins calculated as ruscogenin) and 5 mg of *Ananas comosus* (enzyme activity equal to 1.25 GDU). All substances tested were dissolved in 1 mL of Dulbecco’s Modified Eagle’s Medium (DMEM, Merck Life Science, Rome, Italy) without phenol red and supplemented with 2 mM L-glutamine (Merck Life Science, Rome, Italy), and 1% penicillin–streptomycin (Merck Life Science, Rome, Italy) for all analyses. Vesvein was prepared by mixing all the individual agents and then dissolving them with 1 mL of DMEM without phenol red and FBS and supplemented with 2 mM L-glutamine (Merck Life Science, Rome, Italy), and 1% penicillin–streptomycin (Merck Life Science, Rome, Italy) for all analyses. In addition, Vesvein Double Administration (corresponding to twice the concentration of the standard formulation) is utilised to explore potential dose–response relationships and simulate a higher exposure scenario, such as repeated administration or inter-individual variability in absorption. This approach is in accordance with standard in vitro dose selection practices. The primary objective is to assess whether the biological effects observed are proportional to the level of exposure.

The 96 mM KCl (Merck Life Science, Rome, Italy) solution was prepared using the same culture medium as the other agents under study [10].

### 4.3. Cell Cultures

Human intestinal epithelial cells, Caco-2, purchased from the American Type Culture Collection (ATCC, Manassas, VA, USA), were cultured in Dulbecco’s Modified Eagle’s Medium/Nutrient F-12 Ham’s (DMEM-F12, Merck Life Science, Rome, Italy) containing 10% foetal bovine serum (FBS, Merck Life Science, Rome, Italy), 2 mM L-glutamine (Merck Life Science, Rome, Italy) and 1% penicillin–streptomycin (Merck Life Science, Rome, Italy) and kept in an incubator at 37 °C and 5% CO_2_ [75]. For the experiments, cells were used at passage numbers between 26 and 32 to preserve the integrity of paracellular permeability and transport properties [76], thereby maintaining similarity with the intestinal absorption mechanism and mimicking what occurs after oral intake in humans. According to the experimental protocol, the cells were plated at 1 × 10^4^ cells on 96-well plates to study cell viability using an MTT-based In Vitro Toxicology Assay Kit (Merck Life Science, Rome, Italy) and reactive oxygen species (ROS) production. In addition, 2 × 10^4^ cells were plated on 6.5 mm Transwell^®^ (Corning^®^ Costar^®^, Merck Life Science, Rome, Italy) with a 0.4 μm pore polycarbonate membrane insert (Corning^®^ Costar^®^, Merck Life Science, Rome, Italy) in a 24-well plate to perform the absorption and integrity analyses [77]. From a physiological standpoint, oral compounds typically transit through the small intestine, the primary site of nutrient and drug absorption, within a time window of approximately 2 to 6 h after ingestion, depending on individual gastrointestinal motility and formulation type [78,79]. Before treatment, the cells were placed in DMEM without red phenol (Merck Life Science, Rome, Italy) supplemented with 0.5% FBS (Merck Life Science, Rome, Italy), 2 mM L-glutamine (Merck Life Science, Rome, Italy), and 1% penicillin–streptomycin (Merck Life Science, Rome, Italy) for 8h to synchronise them and then incubated at 37 °C with 5% CO_2_.

Human umbilical vein endothelial cells (HUVECs) purchased from ATCC (Manassas, VA, USA) were cultured in 0.1% gelatin-coated flask with Endothelial Growth Medium-2 (EGM-2) containing 2% FBS, 0.04% hydrocortisone, 0.4% hFGF-B, 0.1% VEGF, 0.1% R3-IGF-1, 0.1% ascorbic acid, 0.1% hEGF, 0.1% GA-1000, 0.1% heparin (all from Lonza, Walkersville, MD, USA) and maintained at 37 °C and 5% CO_2_ as previously described [80]. HUVECs were incubated at 37 °C in a 95% humidified atmosphere with 5% CO_2_ until passage 3 to 6 before being used in the experiments [80]. Therefore, 3 x 10^4^ cells were placed in a 96-well plate in complete growth medium to perform cell viability analysis by MTT test and ROS production analysis. In addition, 7 × 10^4^ cells/well were plated for 48 h in 24-well plates previously coated with Matrigel (BD Biosciences; incubated for 30 min at 37 °C) to analyse the mechanisms involved in venous insufficiency [81]. Specifically, cells placed on Matrigel were treated with KCl to simulate the characteristics of vessel physiology during venous insufficiency, resulting from increased ECM destruction and elevated venous pressure [10].

### 4.4. Intestinal Barrier In Vitro Model

For the study of the substances under investigation, an in vitro intestinal barrier model was created using the Transwell^®^ system, following a standard protocol reported in the literature [82] and approved by European Medicines Agency (EMA) and the Food and Drug Administration (FDA) to predict the absorption, metabolism and bioavailability of different substances after oral intake in humans [83,84]. According to EMA and FDA guidelines, the internal standardisation of the Caco-2 cell line includes permeability studies for model compounds representing a range of in vivo human intestinal absorption, with, respectively, low (fa < 50%), moderate % fraction dose absorbed in humans (fa = 50–84%), and high permeability (fa ≥ 85%) [85,86]. Numerous studies have shown that permeability values estimated with this model correlate well with in vivo human absorption data for many drugs and chemicals [86,87,88,89,90]. Briefly, Caco-2 cells, plated as described above, were maintained in a complete culture medium, changing it every other day on the basal and apical sides for 21 days before the simulations [91]. Throughout the maturation period, TEER values were evaluated by EVOM3, coupled with STX2 rod electrodes (World Precision Instruments, Sarasota, FL, USA), to assess the formation of mature intestinal epithelia and a proper paracellular mechanism. Absorption analysis commenced on day 21, when TEER values were ≥400 Ω∙cm^2^ [92]. Before stimulation, the culture medium on the apical side was adjusted to pH 6.5, the pH of the lumen of the small intestine, while the pH 7.4 on the basolateral side represented blood [76]. Under these experimental conditions, substances were systematically introduced into the apical environment within a temporal framework ranging from 1 to 6 h. At each specified time interval, intestinal permeability was evaluated by applying 0.04% fluorescein (Merck Life Science, Milan, Italy), a fluorescent marker dye utilised to quantify transepithelial transport [93]. The amount of fluorescein transported was measured at 37 °C for 40 min by incubating Caco-2 cells at the above concentration (apical pH, 6.0; basolateral pH, 7.4). Fluorescence was detected with a fluorescence spectrophotometer (Infinite 200 Pro MPlex, Tecan, Männedorf, Switzerland) at excitation/emission wavelengths of 490/514 nm. The cells were stimulated with all substances from 2 to 6 h before subsequent analyses, including the absorption rate analysis. The results are expressed as the proportion of the original amount that permeated through the cells. The permeation rate [nmol min (mg protein)], J, was calculated following [94]:

J = Jmax [C]/(Kt + [C])

where

C: the initial concentration of fluorescein.
Jmax: the maximum permeation rate.
Kt: the Michaelis–Menten constant.
Results are expressed as the means ± SD (%).

### 4.5. Vein In Vitro Model

To establish the effects of substances on the vein, 7 × 10^4^ HUVECs were placed into a 24-well plate coated with Matrigel substrate. A proper maturation time for the vein monolayer was determined by measuring TEER using an EVOM3 device coupled with STX2 chopstick electrodes (World Precision Instruments, Sarasota, FL, USA), until the cells reached a threshold of 20 Ω∙cm^2^ [80].

### 4.6. Experimental Protocol

This investigation was systematically partitioned into two distinct phases: an intestinal phase and a venous phase. As an initial endeavour within the intestinal phase, the assessment of cell viability (utilising the MTT assay) and the quantification of ROS production (employing Cytochrome C reduction) in intestinal cells subjected to treatment with Diosmin/Hesperidin, *Ruscus aculeatus* L., *Ananas comosus* extract, Bromelain, and their combination over a period ranging from 1 to 6 h was conducted; furthermore, the combination above was also administered at a dosage that was twice the standard amount. Subsequently, the examination of the absorption and transport mechanisms of both single- and double-dose formulations, as well as individual agents, was performed through in vitro reconstruction of the intestinal barrier utilising a three-dimensional model, which has been validated in existing literature [95] and approved by the EMA and FDA [82,83]. Measurements of TEER values, absorption analysis and Tigh Junction (TJ) determination were conducted on the 3D intestinal in vitro model. In the venous phase, intestinal metabolite was collected from the basolateral layer of the Transwell^®^ and used to treat the 3D venous model created by seeding HUVECs onto a layer of Matrigel. The in vitro vein model was treated with all test substances for 24 h and then damaged for 15 min with 96 mM KCl [10] to analyse the degree of cell viability, endothelial cell metabolic activity, ATP production, and oxidative/inflammatory status, which are essential for proper vein function. In addition, all mechanisms involved in the processes of maintaining vessel tone were evaluated by assessing elastin following vessel contraction/relaxation movements, vessel wall permeability by analysing tissue integrity (measurement of TEER), and the leading promoters of vessel permeability such as endothelin-1 and eNOS resulting in the production of NO as a vasodilating agent. Finally, the ability of the agents under investigation to maintain vessel architecture was analysed by evaluating the significant cadherins involved, such as VEGF and MMP-9, by Western blot analysis.

The study samples were tested with a known commercial product for all analyses to compare their biological effects in both districts.

### 4.7. Cell Viability

The MTT (3-(4,5-dimethylthiazol-2-yl)-2,5-diphenyltetrazolium bromide) test was performed according to a standard protocol [96] to exclude cytotoxic effects. Briefly, after stimulations, both cell types were incubated with 1% MTT dye (Merck Life Science, Milan, Italy) in white DMEM for 2h at 37 °C in an incubator. Purple formazan crystals were dissolved in an equal volume of solubilising solution. Cell viability was determined by measuring the absorbance at 570 nm with correction at 650 nm, using a spectrometer (Infinite 200 Pro MPlex, Tecan, Milan, Italy), and calculated by comparing the results with control cells without any stimulus (baseline 0%).

### 4.8. Reactive Oxygen Species Production

Quantification of superoxide anion release was obtained following a standard protocol based on cytochrome C reduction [97], and the absorbance in the culture supernatants was measured at 550 nm using a spectrophotometer (Infinite 200 Pro MPlex, Tecan, Männedorf, Switzerland). Specifically, 100 μL of cytochrome C (Merck Life Science, Milan, Italy) was added to all wells, while 100 μL of superoxide dismutase (Merck Life Science, Milan, Italy) and 100 μL of cytochrome C were added to the empty wells; the plate was then incubated for 30 min. The O_2_ rate was expressed as the mean ± SD (%) of nanomoles of reduced cytochrome C per microgram of protein compared to the control (line 0).

### 4.9. TJs Analysis

The lysates derived from Caco-2 cells were utilised to assess the concentration of occludin utilising the Human Occludin (OCLN) ELISA Kit (MyBiosource, San Diego, CA, USA), the level of claudin-1 via the ELISA Kit (Cusabio Technology LCC, Houston, TX, USA), and the measurement of Zonula Occludens (ZO)-1 through the human tight junction protein 1 (TJP1) ELISA kit (MyBiosource, San Diego, CA, USA), following the guidelines provided by the manufacturer [98]. The absorbance was quantified utilising a Tecan spectrophotometer at a wavelength of 450 nm. The data were derived by juxtaposing them against the standard curve, which ranged from 0 to 1500 pg/mL for occludin and from 0 to 1000 pg/mL for both claudin-1 and ZO-1. The findings were articulated as a percentage (%) relative to the control (0 line), which consisted of five distinct experiments executed in triplicate.

### 4.10. Determination of ATP Production

After each stimulation, the medium was removed, and the cells were promptly treated with the constituents of the ATP assay kit (nucleotide-releasing buffer, ATP monitoring enzyme, enzyme reconstitution buffer, and ATP), adhering strictly to the manufacturer’s specifications. Luminescence was quantified one min after the introduction of the ATP monitoring enzyme, utilising a multilabel plate reader (Infinite 200 Pro MPlex, Tecan, Männedorf, Switzerland), and the luminescence data were articulated as means ± SD% of µmol of ATP/g protein [99].

### 4.11. SOD ELISA Kit

SOD levels were evaluated using a commercially available Superoxide Dismutase Assay Kit (Cayman Chemical, Ann Arbor, MI, USA), which detects Cu/Zn, Mn, and Fe SOD [100]. The standard curve, with a range of 0.05–0.005 U/mL, was utilised for the evaluation of SOD levels in cell lysates. The collection of total cell lysates was performed using cold PBS1x. The samples’ absorbances were measured at a wavelength of 480 nm using a spectrometer (Infinite 200 Pro MPlex, Tecan, Männedorf, Switzerland). The results are presented as the mean ± standard deviation (SD) percentage difference compared to the control group.

### 4.12. Tumour Necrosis Factor α (TNF-α) Assay Kit

According to the manufacturer’s instructions, the TNF-α concentration in the supernatant of HUVECs was determined using the TNF-α ELISA kit (Merck Life Sciences, Milan, Italy). The optical density at 450 nm was measured using a plate reader (Infinite 200 Pro MPlex, Tecan, Männedorf, Switzerland); the colour intensity (OD) and the concentration of the standard (ranging from 0 to 6000 pg/mL) were correlated by applying standard curves [101]. The results of five independent tests, conducted in triplicate, were presented as the mean ± SD (%) versus the control (0 lines).

### 4.13. Interleukin 1β (IL-1β) Assay Kit

IL-1β present in HUVEC lysates was measured according to the manufacturer’s guidelines, using an IL-1β ELISA kit (R&D Systems, Minneapolis, MN, USA). The absorbance was assessed using a microplate reader (Infinite 200 Pro MPlex, Tecan, Männedorf, Switzerland) at a wavelength of 450 nm, with a correction implemented at 570 nm. The quantification of IL-1β involved comparing the sample readings against the standard curve that was generated [102].

### 4.14. Interleukin 6 Assay Kit

An IL-6 ELISA kit (eBioscience, San Diego, CA, USA) was utilised to quantify the level of IL-6 present in HUVEC cell lysates, adhering to the manufacturer’s guidelines [103]. The Infinite 200 Pro MPlex spectrometer (Tecan, Männedorf, Switzerland) recorded the greatest absorption wavelength at 450 nm. The results obtained were expressed as a percentage of the control (line 0, untreated cells) and the concentration was represented as pg/mL on a standard curve (0.078 to 5 pg/mL). The result graph is reported in Appendix A.

### 4.15. Interleukin 8 Assay Kit

IL-8 present in HUVEC lysates was measured according to the manufacturer’s guidelines, using an IL-8 ELISA kit—Quantikine (R&D Systems, Minneapolis, MN, USA). The absorbance was assessed using a microplate reader (Infinite 200 Pro MPlex, Tecan, Männedorf, Switzerland) at a wavelength of 450 nm, with a correction implemented at 570 nm. The quantification of IL-8 involved comparing the sample readings against the standard curve that was generated (range 31.2–2000 pg/mL). The result graph is reported in Appendix A.

### 4.16. Human Elastin ELISA Kit

The Human Elastin ELISA kit (MyBiosource, San Diego, CA, USA) was used to quantify elastin levels in HUVEC cell lysates, following the protocols provided by the manufacturer. In summary, 100 µL of the sample was dispensed into a well pre-coated with a specific antibody, followed by a 2 h incubation period at 37 °C. Upon the conclusion of the incubation period, the contents of the wells were discarded, and 100 µL of Biotin antibody was added for an additional 1 h incubation at 37 °C. Following this incubation, the well contents were removed, and the wells underwent a thorough washing procedure three times with a 1x Wash Solution before adding 100 µL of HRP-avidin. After a subsequent incubation of 1 h at 37 °C, the plate was subjected to five wash cycles. Then, 90 µL of TMB Substrate was added to the wells for a 15–30 min incubation at 37 °C. After completing this step, 50 µL of Stop Solution was added to each well, and the absorbance of the plate was measured at 450 nm using a spectrophotometer (Infinite 200 Pro MPlex, Tecan, Männedorf, Switzerland). The results were derived by juxtaposing the obtained data with the standard curve (0–200 pg/mL). They were articulated as a percentage (%) relative to the control (line 0) derived from five independent experiments conducted in triplicate.

### 4.17. Human Endothelin-1 ELISA Kit

Human Endothelin-1 (ET-1) ELISA Kit (ThermoFisher, Waltham, MA, USA) was used according to the manufacturer’s instructions to quantify the amount of Endothelin-1 in the supernatant of HUVECs. Briefly, 50 μL of samples were dispensed into wells, and the plate was incubated at room temperature for 1h. At the end of incubation, the material in the wells was removed, and the wells were washed four times with 1x wash solution. Then, 50 μL of Endothelin-1 Conjugate was added to the wells. After a 1 h incubation, wells were washed four times, and 100 μL of TMB Substrate Solution was added to each well. After 30 min of incubation at room temperature, 50 μL of Stop Solution was added to the well, and the plate was read at 450 nm using a spectrophotometer (Infinite 200 Pro MPlex, Tecan, Männedorf, Switzerland). The results were obtained by comparing the data with the standard curve, which ranged from 0 to 100 pg/mL. They were expressed as a percentage (%) compared to the control (line 0) from five independent experiments performed in triplicate.

### 4.18. Human eNOS/NOS3 ELISA Kit

eNOS concentration was measured following the manufacturer’s instructions of DuoSet ELISA kit for Human eNOS (R&D Systems, Minneapolis, MN, USA) in HUVECs. Using an Infinite 200 Pro-MPlex spectrometer (Tecan, Männedorf, Switzerland), the absorbance of the samples was measured at 450 nm. The results were displayed as a percentage (%) normalised to the untreated samples, based on the standard curve (78.1–5000 pg/mL) created using a standard. The results were presented as mean ± SD (%) compared to the control (line 0) after five different tests were performed in duplicate.

### 4.19. Nitric Oxyde Production

Following the stimulation procedures, Griess’ reagent (Promega, Madison, WI, USA) was introduced to the supernatants derived from HUVECs to evaluate the production of NO [103]. After stimulation, 50 μL of supernatant was extracted from each well and transferred into a new 96-well plate containing 50 μL of sulfanilamide solution. The plate was incubated at 37 °C in a dark environment for 10 min. In the concluding step, 50 μL of N-1-naphthyl ethylenediamine dihydrochloride (NED solution) was added to each well, followed by an additional incubation period of 10 min at 37 °C in the dark. Upon completion of the incubation, a spectrophotometric measurement was conducted using an Infinite 200 Pro MPlex spectrophotometer (Tecan, Männedorf, Switzerland) at a wavelength range of 520–550 nm over a 30- min time frame. This assay can detect NO^2−^, recognised as one of nitric oxide’s most enduring and least volatile derivatives. The results are expressed as a percentage (%) normalised to the untreated control samples, utilising a standard nitrate calibration curve ranging from 0 to 100 µM.

### 4.20. Western Blot Analysis

At the end of each stimulation, keratinocytes were rinsed with cold 1× PBS (Merck Life Science, Rome, Italy) and lysed using Complete Tablet Buffer (Roche, Basel, Switzerland) enriched with 2 mM sodium orthovanadate (Na_3_VO_4_), 1 mM phenylmethanesulfonylfluoride (PMSF) (Merck Life Science, Rome, Italy), a 1:50 phosphatase inhibitor mix (Merck Life Science, Rome, Italy), and a 1:200 protease inhibitor mix (Merck Life Science, Rome, Italy) to yield a total protein extract that underwent laboratory analysis: a 1:50 phosphatase inhibitor mix (Merck Life Science, Rome, Italy) and a 1:200 protease inhibitor mix (Merck Life Science, Rome, Italy) to produce a total protein extract that was centrifuged at 14,000× *g* for 20 min at 4 °C. Subsequently, 40 µg of protein per extract was applied to 8% and 12% SDS-PAGE gels and transferred onto a polyvinylidene difluoride (PVDF) membrane, which was incubated overnight with specific primary antibodies such as MMP9 (1:500, Santa Cruz, CA, USA) and VEGF (1:500, Santa Cruz, CA, USA). The expression levels of all proteins were normalised and confirmed by detecting β-actin (1:5000, Merck Life Science, Rome, Italy) and presented as mean ± SD (%) relative to the control value (line 0).

### 4.21. Statistical Analysis

One-way analysis of variance (ANOVA) and Bonferroni post hoc tests were used to process the data acquired using Prism GraphPad statistical software 9.4.1. A two-tailed Student’s *t*-test was used to compare the two groups. A two-way ANOVA was conducted to evaluate multiple group comparisons, followed by a two-sided Dunnett post hoc test. The mean ± SD of at least five independent (biological replicates, N = 5) experiments performed in triplicate (technical replicates, n = 3) was used to express all results.

## 5. Conclusions

This study’s findings indicate that the novel combination of natural extracts demonstrates effective intestinal permeability and exerts beneficial venous effects without adverse effects, while preserving its biological activity. This in vitro study of the novel combination of *Ruscus aculeatus*, Diosmin/Hesperidin, Bromelain, and *Ananas comosus*, which demonstrates that it exerts protective and anti-inflammatory effects on venous cells, suggesting a potential effect against CVD. Findings in intestinal cell models suggest preservation of barrier properties, potentially relevant for a possible future study on the compound’s bioavailability. In addition, the extracts, rich in flavonoids and saponins, show anti-inflammatory and protective effects on the vascular endothelium. However, while these in vitro results provide essential information on the vascular effects of Vesvein, further investigation is needed to confirm these findings.

This study underscores the proposed formulation’s capacity to promote vascular and metabolic homeostasis through a series of complementary biological mechanisms. The results support the hypothesis that the approach may have potential as an innovative multi-target strategy, which warrants further investigation. However, it is important to acknowledge two main limitations. The study did not evaluate the potential synergistic interactions between the components of the formulation, nor did it include specific stability or degradation tests on the final product. Consequently, subsequent research endeavours will seek to elucidate these aspects by appraising the physicochemical and enzymatic stability of the formulation under simulated gastrointestinal conditions, alongside the synergistic contribution of its active constituents to the collective biological effects.

## Figures and Tables

**Table 1:** Sample and dosages tested.

Sample	Tested Dosage
90:10 mixture of Diosmin/Hesperidin	500 mg
Bromelain 2500 GDU/g	75 mg
*Ruscus aculeatus L.*	37.5 mg
*Ananas comosus L.*	5 mg

## Data Availability

The Laboratory of Physiology stores raw data to ensure permanent retention under a secure system. This study’s data are available from the corresponding author upon reasonable request.

## Abbreviations

The following abbreviations are used in this manuscript:

ANOVA	One-way analysis of variance
ATCC	American Type Culture Collection
CVD	Chronic venous disease
DMEM	Dulbecco’s Modified Eagle’s Medium
DMEM-F12	Dulbecco’s Modified Eagle’s Medium/Nutrient F-12 Ham’s
EC	endothelial cells
ECM	extracellular matrix
EGM-2	Endothelial Growth Medium-2
EMA	European Medicines Agency
eNOS	endothelial nitric oxide synthase
FBS	foetal bovine serum
FDA	Food and Drug Administration
IL-1β	Interleukin 1β
MMPs	metalloproteinases
MMP-9	matrix metalloproteinase-9
MPFF	micronised purified flavonoid fraction
MTT	A3-(4,5-Dimethylthiazol-2-yl)-2,5-Diphenyltetrazolium bromide
Na3VO4	sodium orthovanadate
NO	nitric oxide
PMSF	phenylmethanesulfonylfluoride
ROS	radical oxygen species
TEER	transepithelial electrical resistance
TNF-α	tumour necrosis factor-α
TJ	tight junction
HUVEC	Human umbilical vein endothelial cells
VSMCs	vascular smooth muscle cells
VEGF	vascular endothelial growth factor
Vesvein	mix of 450 mg Diosmin, 50 mg Hesperidin, 37.5 mg Ruscus aculeatus L. extract, 5 mg Ananas comosus L. extract, and 75 mg Bromelain
VVs	varicose veins
ZO-1	zona occludens 1
